# A readily available source of serum for use as a precision quality-control material for ionized calcium measurement by ion-selective electrodes

**DOI:** 10.1155/S1463924682000224

**Published:** 1982

**Authors:** J. A. Fyffe

**Affiliations:** 1 University Department of Medicine Royal Infirmary Glasgow G40SF UK; 2 Department of Biochemistry Queen Elizabeth II Hospital Hertfordshire Welwyn Garden City UK


Journal of Automatic Chemistry, Volume 4, Number 2 (April–June 1982), page 79

### A readily available source of serum for use as a precision quality-control material for ionized calcium measurement by ion-selective electrodes

J. A. Fyffe
*University Department of Medicine, Royal Infirmary, Glasgow G4 0SF, UK* *

A simple method for preparing human serum for use as a precision quality-control for ionized calcium using an ion-selective electrode has been described earlier [1]. Human citrate dextrose plasma is readily available from outdated whole blood from blood transfusion, but human serum is difficult to obtain in sufficient quantities for quality-control purposes. The author has examined a calf serum which is commonly used for quality-control purposes and which is more easily obtainable.

### Method

Ionized calcium was measured by a Nova 2 Analyser (manufactured by Nova Biochemical, Boston, Massachusetts, marketed in the UK by American Hospital Supplies [UK] Ltd, Didcot, Oxon).

Calf sera was kindly donated by Gibco Europe Ltd, Paisley, Scotland. Preparation of the quality-control material was as previously described [1] and sample aliquots were stored at −20°C.

### Results

Levels for total calcium, total protein, albumin, pH and osmolality of this calf sera were all within the human reference ranges. Each working day when the instrument was in use (over a period of five months) a sample of the various pools examined were thawed and assayed for ionized calcium, together with the human serum normally used for precision quality-control. This human serum was used as the criteria of quality control for the analysis. Values for the calf sera were recorded and, at the end of the period, statistics were prepared on the results. These are shown in table 1. The author can offer no explanation for the poorer coefficient of variation of pool 2, as no trends were noted in the quality-control graphs. It is clear that the precisions reflected by these results compare well over this extended period with the results already described for human serum.

*Table 1. Values for calf sera and statistics on the results.*

| Pool number | Number of analyses | Mean (mmol/l) | SD | CV (%) |
|---|---|---|---|---|
| 1 | 42 | 1·110 | 0·0568 | 5·12 |
| 2 | 33 | 1·051 | 0·0776 | 7·39 |
| 3 | 55 | 1·078 | 0·0497 | 4·61 |
| 4 | 44 | 1·215 | 0·0595 | 4·10 |
| 5 | 30 | 1·198 | 0·0574 | 4·79 |

### Discussion

The results show that calf sera can replace human sera, which is difficult to obtain, for use as a precision quality-control for ionized calcium.

### Acknowledgement

The author would like to thank Dr N. Cartwright of Gibco Europe Ltd, Paisley, Scotland, for the supply of calf sera.

### Reference

1. Fyffe, J. A., Jenkins, A. S., Bolland, Carol, J., Dryburgh, Frances J. and Gardner, Mary, D., *Annals of Clinical Biochemistry*, **18** (1981), 110–111.

* *Present address: Department of Biochemistry, Queen Elizabeth II Hospital, Welwyn Garden City, Hertfordshire, UK.*

